# Activation of Toll‐like receptor 4 by thyroid hormone triggers abnormal B‐cell activation

**DOI:** 10.1002/iid3.1007

**Published:** 2023-09-29

**Authors:** Jie Wang, Guo‐Qing Li, Shu Liu, Jing‐Jing Miao, Qi Sun, Wen‐Sha Gu, Xiao‐Ming Mao

**Affiliations:** ^1^ Department of Endocrinology, Nanjing First Hospital Nanjing Medical University Nanjing China

**Keywords:** B cells, Graves' disease, thyroid hormone, Toll‐like receptor 4

## Abstract

**Objective:**

Breakdown of tolerance and abnormal activation of B cells is an important mechanism in the pathogenesis of Graves' disease (GD). High levels of thyroid hormones (THs) play important roles in GD progression. However, the interactions between THs and abnormal activation of B cells remain elusive. This study aimed to explore the effect of high levels of THs on TLR4 expression and abnormal B cell differentiation.

**Materials and Methods:**

Blood samples were collected from patients with GD and healthy controls (HCs) to evaluate the frequency of B cells, their subsets, and TLR4 expression in B cells. A high‐level T3 mouse model was used to study the interaction between THs and the TLR4 signalling pathway.

**Results:**

We found that the frequencies of CD19^+^, CD19^+^ TLR4^+^, CD19^+^ CD86^+^, and CD19^+^ CD138^+^ B cells were significantly higher, as were the expression levels of MRP8/MRP14 and MRP6 and MRP8, MRP14, and MRP6 messenger RNA (mRNA) in peripheral blood mononuclear cells in patients with GD. In high‐level T3 mice models, the serum MRP8/MRP14 and MRP6 levels and the TLR4 mRNA expression in PBMCs were significantly higher. TLR4 mRNA, protein expression, and cytokines downstream of TLR4, such as myeloid differentiation factor 88 (MyD88) and nuclear transcription factor‐κB, were also increased in mouse spleen mononuclear cells.

**Conclusion:**

The present study indicated that high levels of T3 can induce abnormal differentiation and activation of B cells by promoting TLR4 overexpression and provide novel insights into the roles of THs in the pathogenesis of GD.

## 1 INTRODUCTION

Toll‐like receptors (TLRs) are a family of pattern‐recognition transmembrane receptors that play a role in eliciting innate/adaptive immune responses and triggering chronic inflammation.^1^ TLRs sense organisms ranging from bacteria to fungi, protozoa, and viruses by recognizing the conserved molecular patterns they express (called pathogen‐associated molecular patterns/PAMPs), as well as danger‐associated molecular patterns (DAMPs) secreted by damaged tissues, such as myeloid related protein (MRP) family proteins.^2^ MRP8/MRP14 and MRP6 are important members of the MRP family. The MRP8/MRP14 complex is predominantly expressed in myeloid lineage cells, such as neutrophils, monocytes, and activated macrophages, and the increased expression of these complexes has been demonstrated in a range of inflammatory diseases.^3^ MRP6 is mainly expressed by neutrophils, with low expression levels in lymphocytes and nuclear cells.^4^ The best‐known PAMP is lipopolysaccharide (LPS), which is recognized by TLR4 ^5^ and TLR4, and has also been implicated as a receptor for MRP6 and MRP8/MRP14 complexes. The MRP8/MRP14 complex and MRP6 activate the myeloid differentiation factor 88 (MyD88)‐TLR4 signaling pathway, trigger activation of the nuclear transcription factor‐κB (NF‐κB) pathway, and induce the release of proinflammatory cytokines, including tumor necrosis factor‐α (TNF‐α) and interleukin‐17 (IL‐17).^6^, ^7^

Onset of autoimmune diseases often relies on the characteristic autoantibody profiles, emphasizing their association with the activation of autoreactive B cells. The breakdown of B‐cell tolerance plays a key role in the pathogenesis of many autoimmune diseases,^8^, ^9^, ^10^, ^11^ and the production of thyroid‐stimulating hormone receptor antibody (TRAb) by activated B cells plays an important role in the pathogenesis of Graves' disease (GD).^12^ TRAbs persistently stimulate thyroid follicular cells, induce thyroid follicular cell hyperplasia, and secrete excessive thyroid hormones (THs). However, the mechanism by which autoreactive B cells escape self‐tolerance checkpoints remains elusive.^13^

Stimulation of B cells via the TLR pathway not only leads to an increase in antibody production but also promotes cytokine production and upregulation of activation markers, thereby increasing the effectiveness of B cells as APCs.^5^, ^14^, ^15^, ^16^ Previous studies have suggested that TLR4 polymorphisms contribute to the pathogenesis of GD,^16^ and over expression of TLR4 may play an important role in the pathogenesis of GD.^7^, ^17^ Further studies have suggested that GD could promote TLR4 expression in B cells, and TLR4 expression was positively correlated with triiodothyronine (T3) levels in patients with untreated GD.^18^ Therefore, THs may be involved in the breakdown of B‐cell tolerance in GD.

Antithyroid drugs (ATDs) have long been used to treat hyperthyroidism; these drugs function by inhibiting thyroid peroxidase, thus blocking TH synthesis. The aim of ATD treatment is to reduce TH levels to the normal range in patients with hyperthyroidism. After 12–18 months of ATD treatment in patients with GD, more than half of the patients may experience a relapse of hyperthyroidism^19^ and long‐term control of THs with ATDs can reduce GD relapse,^20^, ^21^ indicating the important roles of THs in the development and progression of GD. Recently, many small‐molecule TLR4 agonists have been discovered^22^; however, whether breakdown tolerance and abnormal activation of B cells in GD are due to TLR4 overexpression induced by THs is not fully understood.

In the present study, we found B cells abnormalities were accompanied by elevated TLR4 expression in patients with GD. To determine the role of T3 in TLR4 abnormal expression, a high‐level T3 mouse model was used to study the interaction between THs and the TLR4 signalling pathway. We found that high levels of T3 could induce B cell activation via TLR4, resulting in abnormal expression of the cytokines of the TLR4 signaling pathway in B cells. Our findings provide a new perspective on the interactions between the endocrine and immune systems and provide insights into the involvement of THs in the development and progression of GD.

## 2 MATERIALS AND METHODS

### 2.1 Patients

A total of 102 patients with GD and 37 healthy donors were recruited at Nanjing First Hospital between 2020 and 2021. All participants provided an entire medical history. All patients satisfied the diagnostic criteria for GD, including clinically and biochemically verified hyperthyroidism and positive thyrotropin receptor antibodies. The clinical evaluation included patient history, physical examination, and thyroid ultrasonography. Laboratory and diagnostic testing included determination of serum levels of free thyroxine (FT4), T3 (FT3), thyrotropin (TSH), TPOAb, TGAb, and serum levels of thyrotropin receptor antibody (TRAb). Patients with subacute thyroiditis, hyperfunctioning thyroid nodules, iodine hyperthyroidism, drug‐induced hyperthyroidism, or other causes of hyperthyroidism were also excluded. The exclusion criteria for both patients with GD and healthy controls included diabetes, infectious diseases, other chronic diseases, and cancer. Written informed consent was obtained from all patients and healthy donors, which let all participators to know the uses of their peripheral blood and their anonymized information to be published in this article. This study was approved by the local ethics committee of Nanjing First Hospital (No.: DWSY‐2000629).

### 2.2 Mice

C57BL/6J mice (6–8 weeks old) at an initial weight of 20–22 g were purchased from Beijing Weitong Lihua Experimental Animal Technology Co., Ltd., and maintained under specific pathogen‐free conditions (Experimental Animal Center of Nanjing First Hospital, Nanjing Medical University). Animals were bred and housed in individual cages with free access to standard laboratory water and chow. All experimental procedures were approved by the Experimental Animal Ethics Committee of Nanjing Medical University for the use of live animals for teaching and research.

### 2.3 Experimental design and specimen preparation

Mice were randomly assigned to the control, T3, and T3 + TAK‐242 groups with six mice in each group. The T3 group was subcutaneously injected with T3 (5 μg/10 g) (Meilun Biotech Co., Ltd.) every day for 6 weeks. After 5 weeks of T3 treatment, the T3 + TAK‐242 mice were continued T3 treatment and intravenously injected with TAK‐242 (3 mg/kg) (Med Chem Express Co., Ltd.) from the 5th week at once every other day. The control group was subcutaneously injected with the same volume of saline. The mice were tagged with toe clipping and weighed daily before and after administration. The daily intake of feed and water was recorded. One day after the end of the experiments, the mice were killed under chloral hydrate anesthesia, and the blood, bone marrow, spleen, and other tissues were collected for further analyses, as described below.

### 2.4 Sample collection and cell isolation

Peripheral blood samples were collected from patients with GD and healthy donors. Serum TSH, FT3, FT4, TGAb, TPOAb, and TRAb levels were measured using a chemiluminescence assay (Centaur XP automated chemiluminescence immunoassay analyzer; Siemens). Human peripheral blood mononuclear cells (PBMCs) were prepared from fresh peripheral whole blood using Ficoll‐Hypaque density gradient centrifugation (TBD Science). The spleen was removed before being dissociated on a nylon membrane in a petri dish with D‐hanks. The cell suspensions were centrifuged for 5 min at 200*g*, and the cell pellet was resuspended in 3 mL of saline or culture medium supplemented with 10% fetal bovine serum. BM cells from mice were obtained by flushing the femurs of animals with culture medium injected using a 21 gauge needle. The methods used for isolating PBMCs from mice were the same as those used in humans. CD19^+^ B cells in mice were isolated from PBMCs using CD19^+^ B‐cell isolation kits (Miltenyi Biotec).

### 2.5 Histology

Spleen samples were preserved in 10% formalin, dehydrated, embedded in paraffin sectioned (5 μm thick), and stained with hematoxylin and eosin (H&E) solution (Wako) following routine procedures. The sections were examined, and images were captured using a light microscope (Olympus BX43) equipped with a camera (Olympus DP70). A pathological image analysis system was used in the present study.

### 2.6 T3 treatment in vitro

PBMCs in human peripheral blood were isolated and treated with T3 at 10^3^, 10^4^, 10^5^, and 10^6^ pmol/L (Dalian Meilun Biotechnology Co., Ltd.) for 72 h, in the T3 + TAK‐242 group, T3 was administered for 72 h and 1.0 mM TAK‐242 was administered on the last day. The PBMCs were cultured in complete medium 1640 in an incubator (37°C, 5% CO_2_) for 72 h. The solution was changed approximately 3 days later based on the cell growth. The cell suspension was transferred to separate centrifuge tubes, centrifuged at 250*g* for 5 min, and washed three times with PBS before being used in subsequent experiments.

### 2.7 Serum levels of T3, MRP8/14, MRP6, MRP8, and MRP14

Serum was collected from mice and humans and stored at −80°C until use. Serum levels of MRP8/MRP14 and MRP6 were analyzed using enzyme‐linked immunosorbent assay (ELISA) (Cloud‐Clone Corp.). The concentration of T3 in serum was determined by radioimmunoassay, and the concentrations of MRP8 and MRP14 in serum were determined by ELISA (Cloud‐Clone Corp.), which was performed strictly according to the manufacturer's instructions.

### 2.8 Western blot analysis

The spleen tissues were homogenized, and total proteins were extracted using a tissue and cell total protein extraction kit (Jiangsu KeyGEN BioTECH Corp., Ltd.). The protein expression levels of TLR4, MyD88, and NF‐κB p65 were detected by western blot analysis. Briefly, equal amounts of proteins were separated by SDS‐PAGE and transferred onto a nitrocellulose membrane, which was blocked in Tris‐buffered saline with Tween (TBST) containing 5% nonfat dry milk powder. Membranes were then incubated with rabbit polyclonal TLR4 antibody (Servicebio Technology Co., Ltd.), rabbit polyclonal MyD88 antibody (Servicebio Technology Co., Ltd.), mouse monoclonal NF‐κB p65 antibody (Servicebio Technology Co., Ltd.), and mouse monoclonal GAPDH antibody (Servicebio Technology Co., Ltd.). Blots were visualized with goat horseradish peroxidase (HRP)‐conjugated IgG antibody after incubation, and signals were detected using enhanced chemiluminescence (Bio‐Rad).

### 2.9 Quantitative reverse‐transcription polymerase chain reaction (qRT‐PCR)

The total RNA of the B cells, spleen, and blood was extracted using the conventional TRIzol method (Life Technologies). Reverse transcription was performed using the PrimeScript^TM^ RT Reagent Kit (Takara Bio Inc.) with genomic DNA Eraser, in accordance with the manufacturer's instructions for two‐step RT‐PCR: complementary DNA (cDNA) was first synthesized, and then amplified by PCR. The PCR reaction system was as follows: SYBR ® Premix Ex Taq (2×) 10 μL, cDNA 2 μL, 0.8 μL either upstream or downstream primers, ROX Reference Dye II (50×) 0.4 μL, steam sterilization double water 6 μL. The cycle parameters were: 95°C preheating step for 30 s, followed by 40 PCR cycles of 95°C degeneration for 5 s and 60°C annealing/extension for 34 s. Gene expression of TLR4, MRP8, MRP14, and MRP6 in human cells was determined using real‐time RT‐PCR. Gene expression of TLR4, Myd88, NF‐κB, IL‐1β, IL‐6, and TNF‐α in mouse cells was determined by real‐time RT‐PCR. The normalized expression values for each transcript were calculated as the quantity of target gene messenger RNA (mRNA) relative to the quantity of β‐actin mRNA using the $2^{‐∆∆C_{t}}$method.

### 2.10 Flow cytometry

Cell suspensions obtained from the blood, BM, or spleen of humans and mice were subjected to flow cytometry analyses. Human blood cells were incubated with APC‐anti‐CD19, PE‐anti‐CD27, PC7‐anti‐CD138, Percp5.5‐anti‐CD80, PC7‐anti‐CD86, and PE‐anti‐TLR4. BM cells from mice were incubated with FITC‐anti‐B220 antibody. Blood and spleen cells from mice were stained with FITC‐anti‐B220, PC7‐anti‐CD27, PE‐anti‐TLR4, Percp5.5‐anti‐CD69, and Percp5.5‐anti‐CD80. All antibodies were obtained from BD Pharmingen (San Jose), and samples were acquired on a BD Canton‐II flow cytometer and analyzed using FlowJo software.

### 2.11 Statistical analysis

All experiments were repeated at least in triplicate. The test data are expressed as the mean ± standard deviation. The independent sample *t* test was used for normally distributed parameters and the nonparametric Mann–Whitney *U* test for nonnormally distributed data. The rates were compared using the *χ* ^2^ test. Multiple groups of data were compared using a one‐way analysis of variance. All data were statistically analyzed using SPSS 22.0. Statistical significance was set at *p* < .05.

## 3 RESULTS

### 3.1 The expression of TLR4, MRP8/MRP14, and MRP6 of B cell is significantly increased in patients with GD

**Figure 1:**
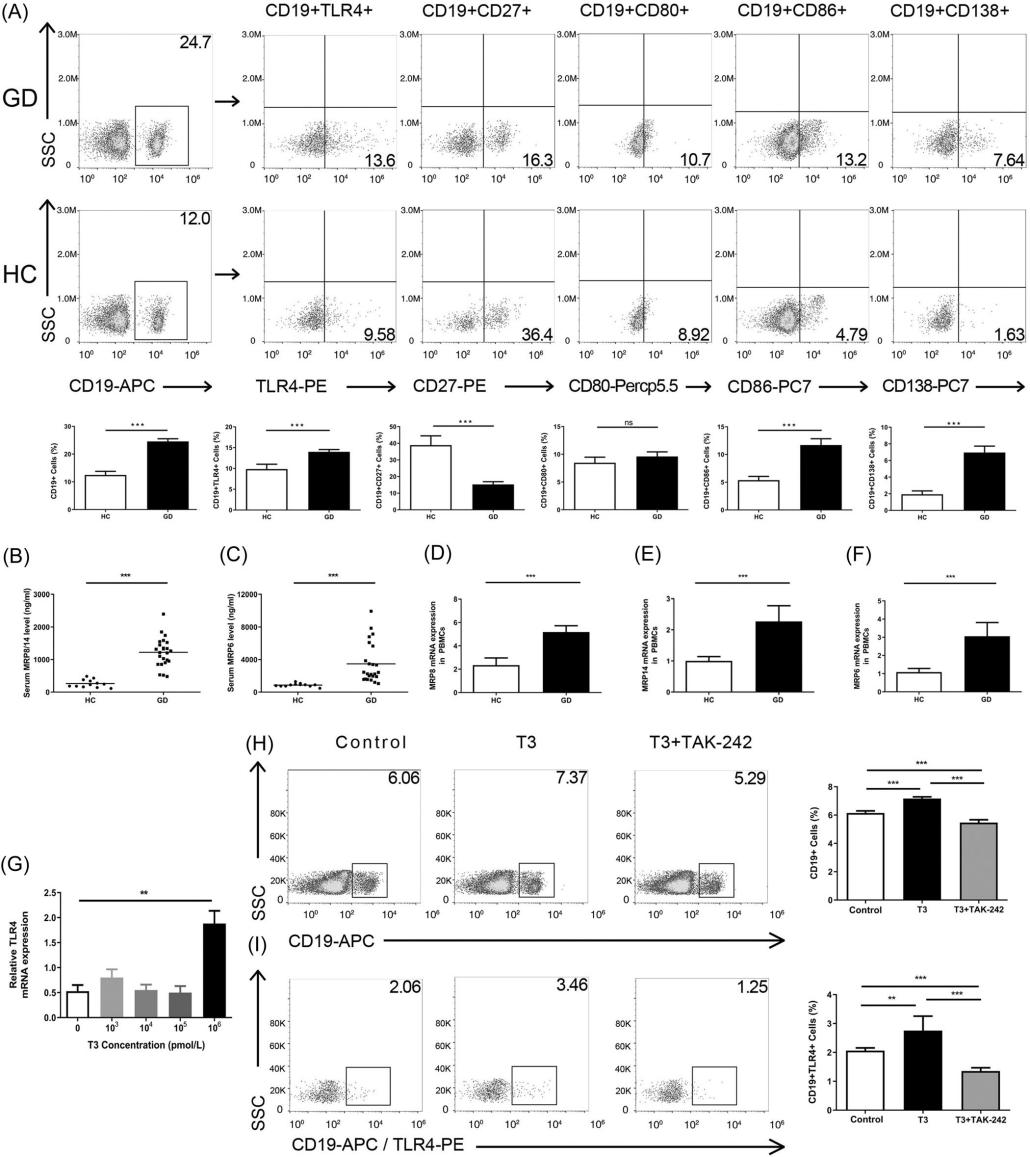
B‐cell expression of TLR4, MRP8/MRP14, and MRP6 is significantly increased in patients with GD. (A) The frequencies of TLR4, CD27, CD80, CD86, and CD138 on CD19^+^ B cells of PBMCs in patients with GD and healthy control (HC). Data are presented as mean ± SD (*n* = 6 independent biological experiments). Statistical significance is assessed by two‐sided independent *t*‐test, ***p < .001. (B, C) The levels of serum MRP8/MRP14 and MRP6 in patients with GD (*n* = 24) and HC.^12^ Data are presented as mean ± SD. Statistical significance is assessed by two‐sided independent *t*‐test, ***p < .001. (D–F) The mRNA expression of MRP8, MRP14, and MRP6 in PBMCs of patients with GD and HC. Data are presented as mean ± SD (*n* = 6 independent biological experiments). Statistical significance is assessed by two‐sided independent *t*‐test, ***p < .001. (G) The mRNA expression of TLR4 in PBMCs of HC after various concentration of T3 stimulation for 72 h. (H) The proportion of CD19^+^ B cells in control, T3 (T3 treated 72 h) and T3 + TAK‐242 groups (T3 treated 72 h and1.0 mM TAK‐242 treated at the last day), Data are presented as mean ± SD (*n* = 3 independent biological experiments). Statistical significance is assessed by two‐sided independent *t*‐test, ***p < .001. (I) The proportion of TLR4 on CD19^+^ B cells in control, T3, and T3 + TAK‐242 groups. Data are presented as mean ± SD (*n* = 3 independent biological experiments). Statistical significance is assessed by two‐sided independent *t*‐test, **p < .01, ***p < .001, respectively. GD, Graves' disease; mRNA, messenger RNA; PBMC, peripheral blood mononuclear cells.

We recruited 102 patients with GD and 37 healthy donors to evaluate the frequencies of B cells, their subsets, and TLR4 expression in B cells. The general clinical characteristics of patients with GD and healthy controls (HCs) are summarized in Table 1. The frequencies of CD19^+^, CD19^+^ TLR4^+^, CD19^+^ CD86^+^, and CD19^+^ CD138^+^ B cells were significantly higher in patients with GD than in HCs; however, the frequency of CD19^+^ CD27^+^ B cells was significantly lower. There was no significant difference in the frequency of CD19^+^ CD80^+^ B cells between the two groups (Figure 1A). The levels of serum MRP8/MRP14 and MRP6, and MRP8, MRP14, and MRP6 mRNA expression in PBMCs were significantly higher in patients with GD than in HCs (Figure 1B–F).

**Table 1:** Clinical and biochemical characteristics.

Groups	Control (*n* = 37)	GD (*n* = 102)	*p*
Female, *n* (%)	26 (70.3)	70 (68.6)	.853
Age (years)	48.32 ± 11.881	45.69 ± 13.49	.295
Duration (months)	‐	7.5 (2,24)	‐
Smoker (%)	5 (13.5)	15 (14.7)	.859
FBG (mmol/L)	5.83 ± 1.29	6.03 ± 1.72	.512
WBC (×10^9^/L)	5.23 ± 1.08	5.45 ± 1.44	.411
ALT (U/L)	18.35 ± 7.13	30.21 ± 19.18	<.001
AST (U/L)	19.16 ± 7.59	23.75 ± 12.01	.033
TSH (mIU/L)	1.6513 (1.0547, 2.7669)	0.0038 (0.0038, 0.4843)	<.001
FT3 (pmol/L)	3.73 (3.45, 4.34)	7.30 (4.42, 15.58)	<.001
FT4 (pmol/L)	13.33 (11.85, 14.77)	17.58 (12.31, 30.73)	.001
TRAb (IU/L)	1.03 (0.8, 1.43)	5.26 (2.24, 19.49)	<.001
TGAb (IU/mL)	1.96 (1.29, 3.57)	16.24 (2.95, 141.02)	.001
TPOAb (IU/mL)	1.35 (0.54, 3.43)	156.67 (13.35, 879.02)	<.001

Because the most important pathological change in GD is the secretion of excessive THs, we treated healthy human PBMCs with various concentrations of T3. The results suggested that low levels of T3 did not affect TLR4 mRNA expression in PBMCs, but high levels of T3 (10^6^ pmmol/L) significantly promoted TLR4 mRNA expression in PBMCs (Figure 1G). Flow cytometry results showed that the frequencies of CD19^+^ and CD19^+^ TLR4^+^ cells were significantly higher after treatment with 10^6^ pmmol/L T3 than in the control group (Figure 1H,I). When we used TAK‐242 to inhibit TLR4 expression, the frequencies of CD19^+^ and CD19^+^ TLR4^+^ cells were significantly lower in the T3 + TAK‐242 group than in the T3 group (Figure 1H,I).

### 3.2 High level of T3 induces TLR4 overexpression in murine PBMCs

**Figure 2:**
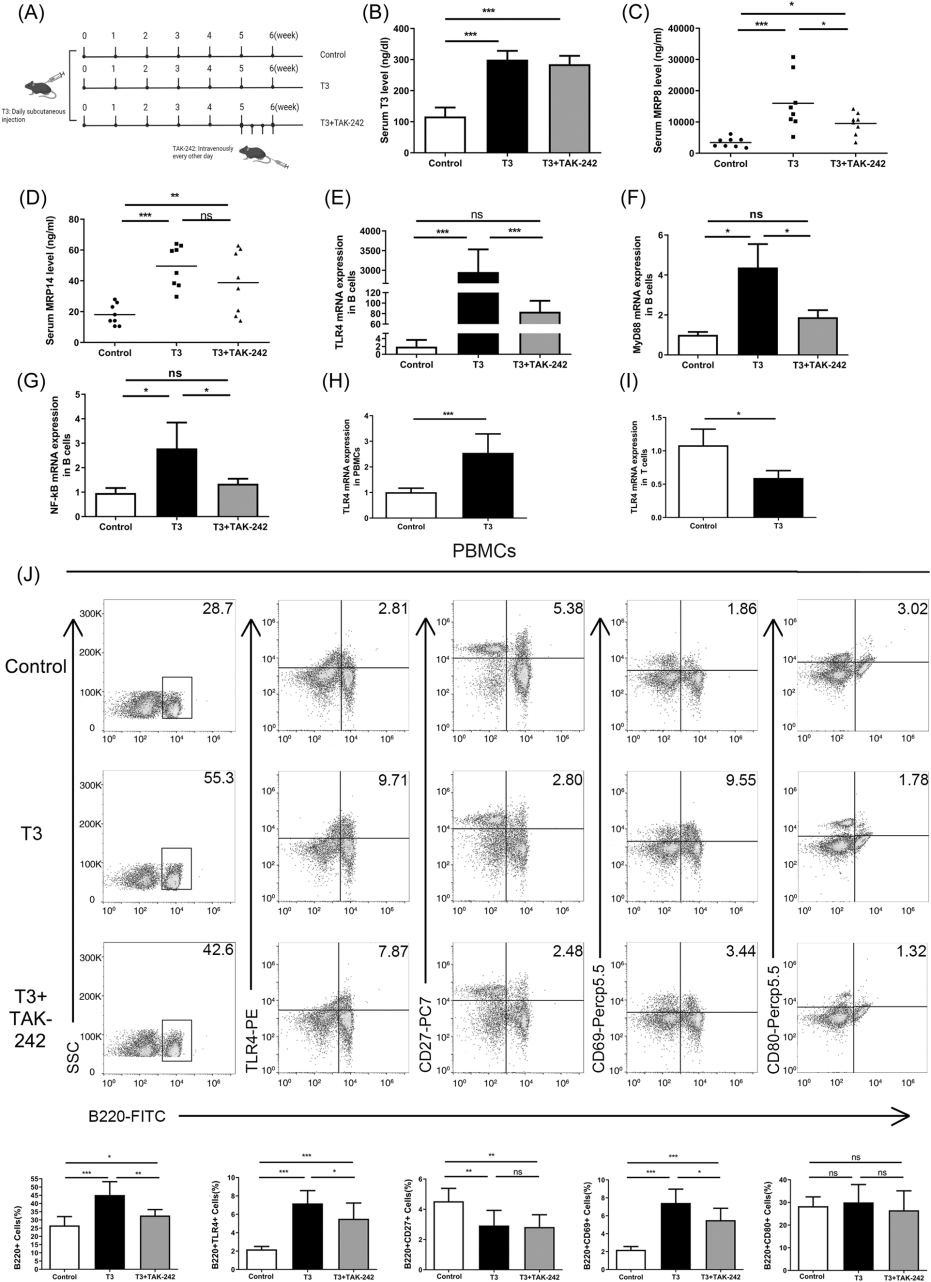
High level of T3 induces TLR4 overexpression in murine PBMCs. (A) The mice were injected subcutaneously with T3 (5 μg/10 g) every day for 6 weeks. After 5 weeks of T3 treatment, the T3 + TAK‐242 mice were continued T3 treatment and intravenously injected with TAK‐242 (3 mg/kg) from the 5th week at once every other day. (B–D) The levels of serum T3 (B), MRP8 (C), and MRP14 (D) for the control, T3, and T3 + TAK‐242 groups of mice after molding. Data are presented as mean ± SD (*n* = 8 independent biological experiments). Statistical significance is assessed by two‐sided independent *t*‐test, *p < .05, **p < .01, ***p < .001, respectively. (E–G) The mRNA expression of TLR4 (E), MyD88 (F), and NF‐κB (G) in B cells for the three groups. (H, I) The mRNA expression of TLR4 in PBMCs (H) and T cells (I) for T3 and control group of the mice. (J) the proportion of TLR4, CD27, CD69, and CD80 on B220+ B cells of PBMCs in control, T3, and T3 + TAK‐242 groups of mice (the top panels). Data are presented as mean ± SD (*n* = 6 independent biological experiments). Statistical significance is assessed by two‐sided independent *t*‐test, *p < .05, ** *p* < .01, ***p < .001, respectively. mRNA, messenger RNA; NF‐κB, nuclear factor‐kappaB; PBMC, peripheral blood mononuclear cells; TLR4, Toll‐like receptor 4.

To confirm the effects of high T3 levels on TLR4, MRP8/MRP14, and MRP6 expression, we used a high‐level T3 mouse model (Figure 2A). After 6 weeks of T3 (5 μg/10 g/d) treatment, the level of T3 was significantly higher than that in the control group, but did not significantly differ between the T3 and T3 + TAK‐242 groups (Figure 2B). The serum MRP8 and MRP14 levels were significantly higher in the T3 group than in the control group (Figure 2C,D). The B‐cell expression levels of TLR4 and its downstream cytokines, such as MyD88 and NF‐κB, was significantly higher in T3‐treated mice than in controls. Compared with the T3 group, the mRNA expression of TLR4, MyD88, and NF‐κB mRNA were significantly reduced in the T3 + TAK‐242 group (Figure 2E–G). Furthermore, TLR4 mRNA expression in PBMCs of the T3 treated group was higher than that in the control group; however, TLR4 mRNA expression in T cells was lower in the T3 treated group than in the control group (Figure 2H,I).

The flow cytometry results suggested that the frequencies of B220 + B cells, B220 + TLR4+, and B220 + CD69^+^ cells were all significantly higher in T3‐treated mice than in the control group, and the frequency of CD27 on B220 + B cells was significantly lower than that in the control group. Compared to the T3 group, the frequencies of B220 + B cells, B220 + TLR4^+^, and B220 + CD69^+^ cells were significantly reduced in the T3‐treated group. However, there was no significant difference in the number of B220+ CD80^+^ B cells between the T3 and control groups (Figure 2J).

### 3.3 High level of T3 induces TLR4, MRP8/MRP14, and MRP6 expression in the spleen of the mouse model

**Figure 3:**
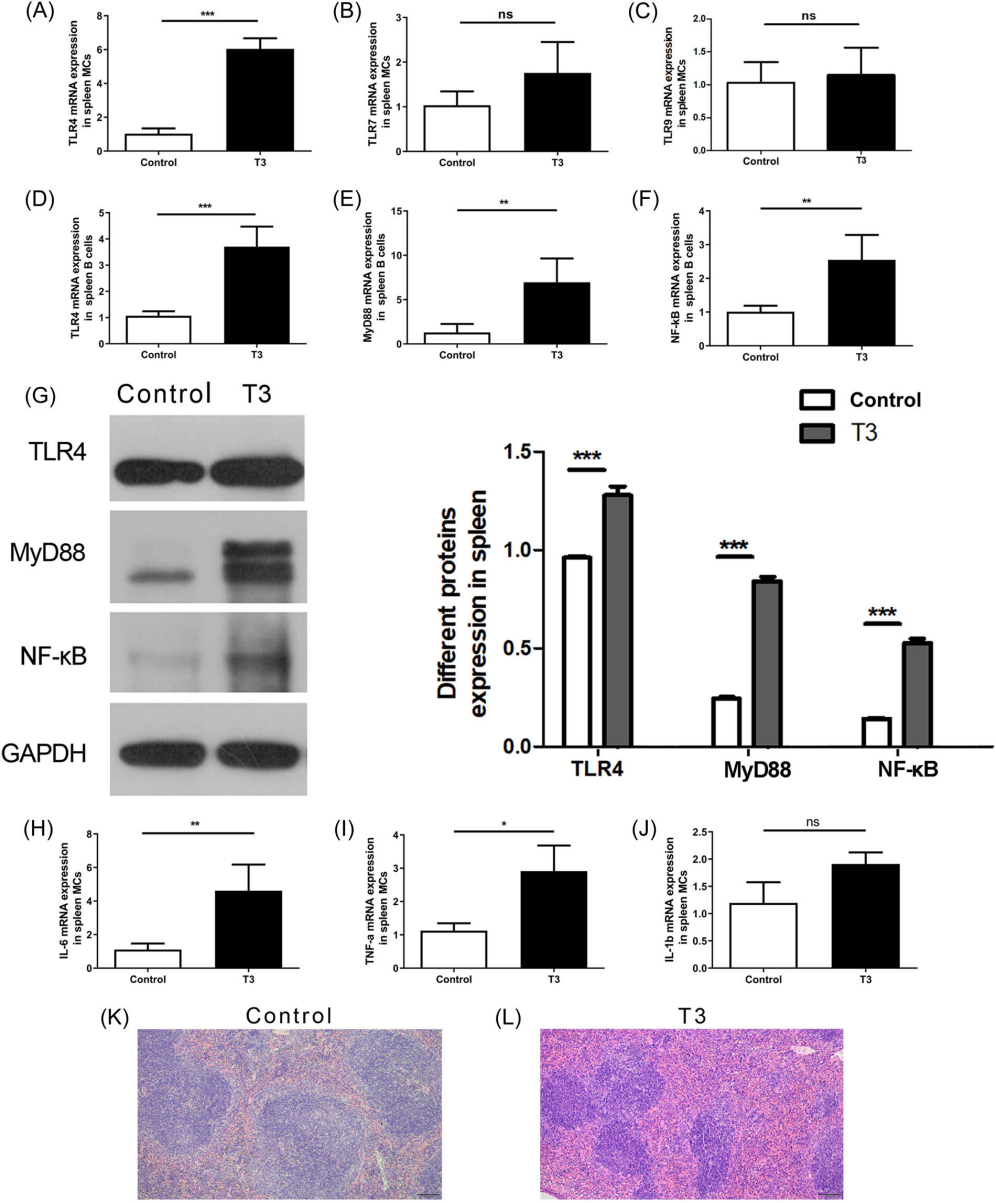
High level of T3 induces TLR4, MRP8/MRP14, and MRP6 expression in the spleen of the mouse model. (A–C) The mRNA expression of TLR4 (A), TLR7 (B), and TLR9 (C) in spleen mononuclear cells (MCs) for control and T3 groups of the mice. (D–F) The mRNA expression of TLR4 (D), MyD88 (E), and NF‐κB (F) in spleen B cells for the two groups. Data are presented as mean ± SD (*n* = 6 independent biological experiments). Statistical significance is assessed by two‐sided independent *t*‐test, **p < .01, ***p < .001, respectively. (G) The protein expression of TLR4, MyD88, and NF‐κB in spleen tissue of the two groups. Data are presented as mean ± SD (*n* = 3 independent biological experiments). Statistical significance is assessed by two‐sided independent *t*‐test, ***p < .001. (H–J) The mRNA expression of IL‐6 (H), TNF‐α (I), and IL‐1b (H) in spleen MCs for the two groups. Data are presented as mean ± SD (*n* = 6 independent biological experiments). Statistical significance is assessed by two‐sided independent *t*‐test, *p < .05, **p < .01, respectively. (L, M) The spleen pathological sections stained with hematoxylin and eosin for the two groups. GAPDH, glyceraldehyde 3‐phosphate dehydrogenase; IL‐6, interleukin 6; mRNA, messenger RNA; TLR4, Toll‐like receptor 4; TNF‐α, tumor necrosis factor‐α.

As expected, TLR4 mRNA expression in mononuclear cells (MCs) of the spleen increased in T3‐treated mice compared to that in the control (Figure 3A). However, TLR7 and TLR9 mRNA expression was not affected by high levels of T3 in spleen MCs (Figure 3B,C). TLR4, MyD88, and NF‐κB mRNA and protein expression in B cells of the spleen were higher than those in T3 treated mice than in the control group, which corresponds with the results seen in humans (Figure 3D–G). Furthermore, IL‐6 and TNF‐α were also increased in spleen MCs of the T3‐treated group compared with the control group (Figure 3H,I), but IL‐1b was not affected by T3 treatment in the spleen MCs (Figure 3J). We noted that the spleen white pulp compartment was expanded and fused in T3‐treated mice compared with that in the control group, as shown on H&E staining (Figure 3K,L).

**Figure 4:**
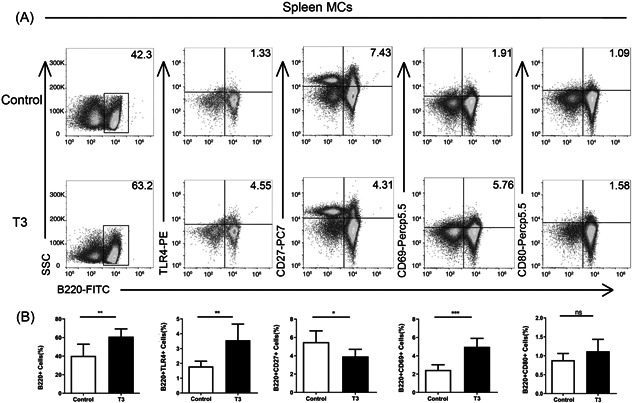
High level of T3 induces B cells abnormal differentiation and activation of B cells in the spleen of the mouse model. (A, B) The frequencies of TLR4, CD27, CD69, and CD80 on B220 + B cells of spleen MCs in control and T3. Data are presented as mean ± SD (*n* = 6 independent biological experiments). Statistical significance is assessed by two‐sided independent *t*‐test, *p < .05, **p < .01, ***p < .001, respectively. MC, mononuclear cells.

The frequencies of B220 + B cells, and their expression of TLR4, and CD69 in spleen MCs were all significantly higher in T3 treated mice than in the control group, and the frequency of CD27 on B220 + B cells was significantly lower in the T3 treated mice than in the control group. However, there was no significant difference in the number of B220 + CD80 + B cells between the T3 and control groups (Figure 4A).

## 4 DISCUSSION

In the current study, we found that TLR4 was upregulated in B cells, and serum MRP8/MRP14 and MRP6 levels and their corresponding mRNA expression in PBMCs were elevated in patients with GD, which agrees with the results of previous studies.^7^, ^17^ We further demonstrated that high levels of T3 treatment in mice could induce the overexpression of TLR4 and its ligands, such as MRP8/MRP14 and MRP6, as well as the downstream molecules of TLR4, such as MyD88 and NF‐κB, and inflammatory cytokines, such as IL‐6 and TNF‐α. The frequency of B cells (CD19^+^ cells) and plasma cells (PCs, CD19^+^ CD138^+^ cells) was increased in treated mice, suggesting that B cells showed abnormal differentiation in patients with GD. The frequency of memory B cells (CD27) decreased in patients with GD, which may be due to the differentiation of memory B cells into PCs. PCs are terminally differentiated B cells that produce antibodies which provide immediate and long‐term protection against pathogens. B cells are targeted because of their key role as enhancers of the immune response in autoimmunity, as they give rise to autoantibody‐producing PCs.^23^ Furthermore, the frequency of CD19^+^ CD86^+^ cells in patients with GD and CD220^+^ CD69^+^ cells in the high‐level T3 mouse models were also increased.

CD69, CD80, and CD86 are all B‐cell activation molecules. CD69 is expressed on most activated immune cells, especially in the early activation process, is used as a general marker for activated immune cells, and is a potentially more sensitive indicator of early subclinical immune responses than a cell‐specific marker.^24^, ^25^ The expression of costimulatory molecules such as CD86 and CD80 on B cells is also augmented by stimulation with TLRs.^26^

The mechanism by which THs induce TLR4, MRP8/MRP14, and MRP6 overexpression is unknown. The THs mainly included T3 and tetraiodothyronine (T4). T4 is a pro‐hormone that is exclusively synthesized and secreted by thyroid follicular cells. T4 enters the circulation to reach target tissues, where it is converted into T3 by type 2 iodothyronine deiodinase (DIO_2_), playing physiological roles in tissues. TH nuclear receptors (TRs) mediate the biological activity of T3 via transcriptional regulation. Two human TR genes, *THRA* and *THRB*, encode three thyroid hormone‐binding receptor isoforms (α1, β1, β2). The transcriptional activity of these TRs is regulated at multiple levels. In addition to being regulated by T3, transcriptional activity is regulated by the TH response elements located on the promoters of T3 target genes, by the developmental‐ and tissue‐dependent expression of TR isoforms, and by a host of nuclear coregulatory proteins.^27^ These nuclear coregulatory proteins modulate the transcriptional activity of TRs in a T3‐dependent manner.^28^ Previous studies have indicated that B cells express both TRα1 and TRα2, whereas TRα2 encodes an orphan nuclear receptor that does not bind T3.^29^ Intrinsic expression of TRα1 and TRα2 gene products is required for the development of a normal B‐cell pool.^30^ B‐cell lymphopoiesis was suppressed by the TRα1 mutation, and mutations in the *THRA* gene in patients could lead to B‐cell deficiency.^31^ We speculate that THs may induce B cell activation, which may be due to the genetic transcription effect of TLR4 promoted by T3. The exact mechanism of T3‐induced TLR4 overexpression requires further investigation.

Although polymorphisms, expression, and activation of TLR7 and TLR9 are associated with GD,^32^, ^33^ unlike TLR4, T3 could not stimulate the overexpression of these genes in mouse spleen MCs. This may be due to the selective expression of genes with T3 stimulation. We found that TLR4 mRNA expression was moderately reduced in dendritic cells expressing TRβ^34^ and iodothyronine deiodonases type 2 and 3 (D2, D3), and exhibited both enzymatic activities with a prevalence of TH inactivation.^35^ Although there is no direct evidence of TR expression in T cells, deficiencies of T3Ra1 and T3Ra2 gene products have been shown to affect spleen cell numbers, and B cells were more severely affected than T cells.^30^

Of course, there are still some limitations to our study. Our study focused on frequencies in B cell clusters and did not detect the expression of CD27, CD69, CD80, CD86, and CD138 genes on B cells. At the same time, the results of the mouse experiment have not been further verified in humans. In addition, our study was limited to the effects of T3 on B cells and ignored the effects of T3 on other immune cells, such as myeloid cells.

In conclusion, our findings indicate that abnormal expression of TLR4 in patients with GD may be induced by high levels of THs, which results in B cell proliferation and activation. These results provide a new perspective on the interactions between the endocrine and immune systems, and provide insight into the involvement of THs in the development and progression of GD.

## AUTHOR CONTRIBUTIONS

**Jie Wang**: Writing—original draft. **Guo‐Qing Li**: Writing—review and editing. **Shu Liu**: Data curation; formal analysis. **Jing‐Jing Miao**: Investigation; methodology. **Qi Sun**: Software; supervision. **Wen‐Sha Gu**: Validation. **Xiao‐Ming Mao**: Conceptualization; project administration; writing—review and editing.

## CONFLICT OF INTEREST STATEMENT

The authors declare no conflict of interest.

## ETHICS STATEMENT

This study was approved by the local ethics committee of Nanjing First Hospital (No.: DWSY‐2000629). All of the experimental procedures used in this study were conducted in accordance with the Experimental Animal Ethics Committee of Nanjing Medical University, Jiangsu Province, China. Written informed consent was obtained from the patients for the using their peripheral blood and their anonymized information to be published in this article.

## Supporting information

Supporting information.

## Data Availability

The data are available from the corresponding author on reasonable request.
